# GameRank: R package for feature selection and construction

**DOI:** 10.1093/bioinformatics/btac552

**Published:** 2022-08-11

**Authors:** Carsten Henneges, Joseph N Paulson

**Affiliations:** Product Development Data Sciences, Genentech Inc., South San Francisco, CA 94080, USA; Product Development Data Sciences, Genentech Inc., South San Francisco, CA 94080, USA; Department of Biochemistry & Molecular Biology, Penn State College of Medicine, Hershey, PA 17033, USA

## Abstract

**Motivation:**

Building calibrated and discriminating predictive models can be developed through the direct optimization of model performance metrics with combinatorial search algorithms. Often, predictive algorithms are desired in clinical settings to identify patients that may be high and low risk. However, due to the large combinatorial search space, these algorithms are slow and do not guarantee the global optimality of their selection.

**Results:**

Here, we present a novel and quick maximum likelihood-based feature selection algorithm, named GameRank. The method is implemented into an R package composed of additional functions to build calibrated and discriminative predictive models.

**Availability and implementation:**

GameRank is available at https://github.com/Genentech/GameRank and released under the MIT License.

## 1 Introduction

Current models used for predicting clinical outcomes of patients should demonstrate three statistical properties: calibration, discrimination and clinical utility. Calibration ensures that predicted and observed outcomes are in agreement. Discrimination reveals if a model can distinguish between outcomes, e.g. patients requiring a therapeutic intervention or not. Clinical utility often requires confirmation by an external body of evidence, for example, a randomized controlled clinical trial that the application of the predictive model leads to superior clinical outcomes (Austin *et al.*, 2020; Crowson *et al.*, 2016; van Calster *et al.*, 2019; Walsh *et al.*, 2017).

Often, measures for calibration are among the first evaluated when building clinical predictive models. For regression-based models, this includes the mean squared error, for binomial and survival models the measures entail percentiles of the mean absolute difference between observed and predicted probabilities, or quantile measures like EC50, EC90 or even $E_{\max}$ and the integrated calibration index (ICI). All of these require either estimation by cross-validation, bootstrap or being evaluated on a hold-out validation dataset.

Finally, a model built on as few clinical variables as possible is often desirable for practical applications may be severely limited by the greater the number of variables. Careful variable selection and variable construction is essential for successful predictive clinical modeling.

Broadly variable selection methodologies can be categorized into three approaches: wrapper algorithms, filter and embedded methods (Guyon and Elisseeff, 2003). Wrapper algorithms perform a combinatorial optimization process by searching according to pre-defined rules and sets of parameters at each step. Well-known search approaches include forward selection, backward selection, and random search. We’ve enabled user-defined model fitting and evaluation functions to be flexibly parameterized to automatically build calibrated and discriminative predictive clinical models.

Filter methods often employ thresholds on statistics estimated from statistical tests or distributional estimation procedures. Examples of statistical tests include parametric or non-parametric tests, such as the *t*-test or Wilcoxon-Rank Sum test, and variable entropy estimation is one approach for a distributional procedure.

Embedded methods include decision trees, random forests, support vector machines with recursive feature elimination, shrinkage estimators, i.e. LASSO, or gradient boosting models where model fitting is intertwined with variable selection. None of these approaches directly aims to optimize calibration measures.

Here, we present an R package consisting of a framework for clinical variable selection that includes a novel algorithm, GameRank, which has been previously applied to building a clinical predictive model for the prediction of chemotherapy tolerability (Harris *et al.*, 2022).

## 2 GameRank algorithm

While most algorithms explore the search space by a strategy, e.g. adding or removing features in a sequential process, until reaching an optimum.

The idea of GameRank is to first explore the search space by evaluating pairs of feature combinations of fixed size against each other. The predictive contribution of each feature can be quantified followinfg multiple combinations are evaluated.

GameRank first generates a *feature learning dataset* by randomly sampling feature combinations of size t and comparing them for r rounds on random 50:50 splits of the input data. One split is used to generate the model for the feature selection, the other is used to evaluate the model. The better model receives a positive score. Note, a model can be better by means of the objective function or by producing a reliable model. The rounds stop if every feature has been evaluated at least k times or after a defined maximum number of iterations.

All of these comparisons are then used to estimate the maximum likelihood (ML) model for ranking individuals by team comparisons published by Huang *et al.* (2008).

The log-likelihood function applied is:

minv-∑i=1mni+logeTi+eTi++eTi-+ni-logeTi-eTi++eTi-

where v is a vector of ranking scores, $n_{i}^{+}$, $n_{i}^{-},$ respectively denote the number of times the $T^{+}$ or $T^{-}$-team wins and m denotes the number of matches.

Due to the ML approach of this ranking model, it is possible to obtain standard errors and confidence intervals per feature, quantifying the uncertainty in the ranking. Using the Delta method, it is also possible to calculate confidence intervals for the strength of any feature selection of interest. This ranking estimate will be asymptotically consistent, such that the size of the feature learning dataset determines the quality of the ranking in the long run.

## 3 R package and its usage

Aside from GameRank, our R package implements a standard set of algorithms for feature selection, including random search. These algorithms make use of at least one training and validation split in determining selections. For all algorithms, two functions need to be provided: one that fits the model to given training data, and one that evaluates the metric on given validation data. We provide standard implementations for these functions for regression, binary response and survival use cases. For binary and survival outcomes, calibration is estimated using methods comparing observed to predicted probabilities, as described in Austin *et al.* (2020) and Crowson *et al.* (2016).

Before variable selection makes sense, a thorough review of each included variable is essential. From our experience, Harris *et al.* (2022), clinical variables can be degenerate, show high distribution skew or may even be multi-modal. Therefore, we provide methods to evaluate variable transformation (sqrt, cube root, log), e.g. if a transformation increases Normality. We provide Box-Cox transformations for regression and binary outcome scenarios and detect multi-modality through Gaussian Mixture-Modeling with automatic model selection via the Akaike Information Criterion. If any distribution is found to be multi-modal, cut points are determined and categorical variables are constructed automatically. For post-processing the selection, we have supplemented the package with functionality to determine influential observations.

## 4 Results

We evaluated the GameRank algorithm on the real-world dataset used in Harris *et al.* (2022) We compared the time, performance as measured by area under the ROC curve and bias between hold-out and validation sets for GameRank, random, forward, backward and the bidirectional selection algorithms for feature selection (Table 1). We observed that GameRank, while being slower than random search and forward selection for very small variable sizes, maintains a very stable runtime independent of the number of features selected. Backward and bidirectional search require long runtimes and even further reduction of the selection problem to 100 input variables for backward selection. Performance-wise backward selection achieved the best result, especially for smaller combinations, however, is unable to run with large >100 variable sets. Random search and GameRank are comparable for larger selection sets. With regards to bias, we can see that GameRank achieves a relatively small and constant bias between the performance on the validation and the predictions on the hold-out set. This is similar to backward selection but achieved with a much shorter computation time.

## 5 Conclusions

The GameRank package is designed to to successfully and efficiently building clinical predictive models. It includes steps for variable construction, variable selection, and model checking. It is supplemented by a novel wrapper algorithm that achieves robust selections with short computation time through a model-based approach. All features are accompanied with examples and all model building steps are described in an easy-to-run vignette.

*Financial Support*: none declared.

*Conflict of Interest*: none declared.

**Table 1.** Benchmark results of GameRank algorithm

*m*	Random	GameRank	Forward	Backward	Bidirectional
5	[1.7, 1.9, 2.2; *n* = 100]	[32.5, 36.3, 39.3; *n* = 100]	[10.2, 45.7, 64.5; *n* = 100]	[923.9, 1153.4, 1377.7; *n* = 76]	[1087.0, 1200.7, 1313.1; *n* = 100]
10	[3.3, 3.4, 3.9; *n* = 100]	[34.5, 36.7, 41.7; *n* = 100]	[34.8, 42.7, 84.2; *n* = 18]	[923.0, 1149.2, 1312.9; *n* = 74]	NA
25	[7.3, 8.1, 12.5; *n* = 99]	[35.4, 36.9, 39.0; *n* = 100]	NA	[853.8, 1113.0, 1362.9; *n* = 78]	NA

*m*	Random	GameRank	Forward	Backward	Bidirectional

5	0.6978 (0.0146; *n* = 100)	0.6349 (0.0514; *n* = 100)	0.7307 (0.0174; *n* = 100)	0.7756 (0.0200; *n* = 76)	0.7383 (0.0181; *n* = 100)
10	0.7307 (0.0118; *n* = 100)	0.6813 (0.0368; *n* = 100)	0.7613 (0.0170; *n* = 18)	0.7759 (0.0201; *n* = 74)	NA
25	0.7787 (0.0190; *n* = 99)	0.7478 (0.0247; *n* = 100)	NA	0.7797 (0.0182; *n* = 78)	NA

*m*	Random	GameRank	Forward	Backward	Bidirectional

5	0.0068 (0.0175; *n* = 100)	0.0035 (0.0253; *n* = 100)	−0.0094 (0.0228; *n* = 100)	0.0136 (0.0247; *n* = 76)	−0.0097 (0.0237; *n* = 100)
10	0.0218 (0.0155; *n* = 100)	0.0036 (0.0217; *n* = 100)	−0.0036 (0.0276; *n* = 18)	0.0136 (0.0250; *n* = 74)	NA
25	0.0544 (0.0227; *n* = 99)	0.0020 (0.0185; *n* = 100)	NA	0.0185 (0.0215; *n* = 78)	NA

*Note*: Time, performance and bias for feature selection algorithms. *m*, size of the feature selection; NA, not available, due to time out (after 3 days of computation) or otherwise. GameRank results are from team size of 10 and 30 rounds being the most stable. *n*, number of iterations successfully completed.
